# Miss Frances Black

**Published:** 1913-03

**Authors:** 


Miss Frances Black, formerly Superintendent of the Buffalo Homoeopathic Hospital, late Superintendent of the Flower Hospital, New York, died of pneumonia February 17.

